# A Recombinant Antibody Against Human DRP1 Serine 616 Phosphorylation Enables Detection of BRAF^V600E^-Associated Mitochondrial Division in Cancer

**DOI:** 10.3390/antib15020038

**Published:** 2026-04-20

**Authors:** Shanon T. Nizard, Yiyang Chen, Madhavika N. Serasinghe, Ruben Fernandez-Rodriguez, Kamrin D. Shultz, Jesminara Khatun, Anthony Mendoza, Jesse D. Gelles, Juan F. Henao-Martinez, Ioana Abraham-Enachescu, Md Abdullah Al Noman, Stella G. Bayiokos, J. Andrew Duty, Shane Meehan, Mihaela Skobe, Jerry Edward Chipuk

**Affiliations:** 1 Laboratory of Mitochondrial Biology in Human Health and Disease, Icahn School of Medicine at Mount Sinai, One Gustave L. Levy Place, New York, NY 10029, USAjesminara.khatun@mssm.edu (J.K.); juan.henao@icahn.mssm.edu (J.F.H.-M.);; 2 Department of Oncological Sciences, Icahn School of Medicine at Mount Sinai, One Gustave L. Levy Place, New York, NY 10029, USA; 3 Mount Sinai Tisch Cancer Center, Icahn School of Medicine at Mount Sinai, One Gustave L. Levy Place, New York, NY 10029, USA; 4 The Graduate School of Biomedical Sciences, Icahn School of Medicine at Mount Sinai, One Gustave L. Levy Place, New York, NY 10029, USA; 5 Laboratory of Metastasis and Lymphatic Research, Icahn School of Medicine at Mount Sinai, One Gustave L. Levy Place, New York, NY 10029, USA; 6 Center for Therapeutic Antibody Development, Icahn School of Medicine at Mount Sinai, One Gustave L. Levy Place, New York, NY 10029, USA; 7 Department of Microbiology, Icahn School of Medicine at Mount Sinai, One Gustave L. Levy Place, New York, NY 10029, USA; 8 Department of Dermatology, Icahn School of Medicine at Mount Sinai, One Gustave L. Levy Place, New York, NY 10029, USA; 9 Department of Dermatopathology, Icahn School of Medicine at Mount Sinai, One Gustave L. Levy Place, New York, NY 10029, USA; 10 The Diabetes, Obesity, and Metabolism Institute, Icahn School of Medicine at Mount Sinai, One Gustave L. Levy Place, New York, NY 10029, USA

**Keywords:** BRAF, cancer, DRP1, melanoma, mitochondrial dynamics, oncogenes

## Abstract

Background/Objectives: Mitochondria are dynamic organelles that continuously undergo balanced cycles of fusion and division to maintain optimal function. Mitochondrial division is mediated by Dynamin-Related Protein 1 (DRP1), a cytosolic large GTPase whose phosphorylation at serine 616 (DRP1-S616Ⓟ) promotes its translocation to the outer mitochondrial membrane and organelle division. Dysregulated mitochondrial division disrupts cellular homeostasis and contributes to disease pathogenesis, including cancer. Our prior work demonstrated that the oncogene-induced mitogen-activated protein kinase (MAPK) pathway constitutively phosphorylates DRP1 at serine 616, which is essential to cellular transformation and correlates with oncogene status in patient tissues. Similarly, DRP1-S616Ⓟ is subject to pharmacologic control by targeted therapies against oncogenic MAPK signaling. Methods: Building upon this foundation, we developed and characterized a recombinant murine monoclonal antibody (referred to as 3G11) with high specificity for human DRP1-S616Ⓟ, raised against a peptide derived from the human DRP1 sequence. Results: Using diverse experimental platforms, we demonstrate the robust utility of 3G11 to detect DRP1-S616Ⓟ in melanoma cell extracts and isolated organelles. Immunofluorescence revealed that pharmacologic inhibition of oncogenic MAPK signaling reduces DRP1-S616Ⓟ levels, which correlates with mitochondrial hyperfusion, while immunohistochemistry showed that elevated DRP1-S616Ⓟ expression in human tissues correlates with BRAF^V600E^ disease. Conclusions: 3G11 is a new recombinant antibody for detecting DRP1-S616Ⓟ and supports studies of mitochondrial division in cancer. Together, these findings establish 3G11 as a specific, versatile, renewable, and cost-effective tool for studying mitochondrial division, with strong potential for clinical applications.

## 1. Introduction

Mitochondria are double-membrane organelles that undergo regulated cycles of fusion and division to maintain cellular and organismal homeostasis [1,2,3]. Dysregulation of these dynamic processes contributes to various pathological conditions, such as inflammation, metabolic disorders, neurodegeneration, and cancer [4,5]. Mitochondrial dynamics are primarily coordinated by large GTPases: Mitofusin 1 (MFN1) and Mitofusin 2 (MFN2) mediate outer mitochondrial membrane (OMM) fusion, while Optic Atrophy 1 (OPA1) controls inner mitochondrial membrane (IMM) fusion [6]. Mitochondrial division is mediated by Dynamin Related Protein 1 (DRP1), a monomeric cytosolic protein that translocates to the OMM upon activation, binds to adaptor proteins (e.g., Mitochondrial Fragmentation Factor, MFF), and oligomerizes to mediate GTP-dependent scission of both the OMM and IMM [2].

Mitochondrial fusion ensures the proper exchange of lipids, metabolites, mtDNA, and proteins between mitochondria, which is essential for maintaining organelle function [1]. Mitochondrial division is critical for the distribution of mitochondria to daughter cells during mitosis, subcellular localization of mitochondria to meet local metabolic demands, and facilitating mitochondrial quality control [2]. The regulation of DRP1-dependent mitochondrial division is a central regulator of disease biology across multiple organ systems. When DRP1 is hyperactivated or suppressed, it directly impacts metabolism, apoptosis, inflammation, and cell fate. As such, DRP1 is subject to a variety of post-translational mechanisms (e.g., phosphorylation) that regulate DRP1 activation, membrane translocation, and oligomer stabilization [7].

The Mitogen-Activated Protein Kinase (MAPK) pathway is a master regulator of proliferation, differentiation, and survival that directly controls DRP1 function [8]. Previous studies suggest a mechanistic connection between oncogene-induced MAPK (e.g., RAS^Q61R^, BRAF^V600E^) signaling and mitochondrial dynamics. Specifically, ERK1/2 (p42/p44) phosphorylates DRP1 at serine 616 (DRP1-S616Ⓟ), thus activating DRP1 translocation to the OMM and mitochondrial division [9]. Oncogene-induced chronic mitochondrial division leads to impaired mitochondrial function, characterized by reduced oxidative phosphorylation and an increased reliance on alternative metabolic pathways. The literature suggests that DRP1-dependent mitochondrial division is essential to cancer mechanisms as silencing DRP1 inhibits transformation [9]. Furthermore, pharmacological inhibition of MAPK signaling in RAS and BRAF mutant human cancer models eliminates DRP1-S616Ⓟ, resulting in hyperfused mitochondria, reactivation of mitochondrial function, and sensitization to cell death [9]. DRP1-S616Ⓟ is observed in tumors with oncogenic MAPK mutations and is suggested to support the development and growth of breast, colon, glioblastoma, lung, pancreatic, and skin cancers [10,11,12,13,14,15].

In the majority of cutaneous melanomas, the BRAF^V600E^ mutation occurs early in pathogenesis but is insufficient to induce malignancy and requires additional events to initiate disease [16,17]. One downstream event subsequent to BRAF^V600E^-induced transformation is sustained DRP1-S616Ⓟ, suggesting chronic mitochondrial division may be a robust biomarker for BRAF^V600E^ melanoma and progression. Indeed, DRP1-S616Ⓟ status dichotomized with BRAF^V600E^ and was detected in ~80% of nevi that subsequently progressed to melanoma, where it persisted throughout the melanomagenesis process [18]. This persistence highlights the potential for DRP1-S616Ⓟ as a reliable indicator for disease initiation and a promising tool for improving our understanding of melanoma biology. However, most antibodies raised against DRP1-S616Ⓟ are traditional monoclonals with undefined variable heavy/light regions (VH/L), restricted by commercial availability and cost, and the early generation hybridomas are subject to genetic drift, potentially impacting their specificity. To circumvent these constraints, we developed a recombinant monoclonal DRP1-S616Ⓟ antibody, derived from a human DRP1 peptide, referred to as 3G11. We validated the 3G11 antibody using a range of techniques, including immunoblotting, immunofluorescence, and immunohistochemistry in melanoma models and patient tissues. These data support the specificity of 3G11 for DRP1-S616Ⓟ and highlight its potential utility in detecting oncogene-induced chronic mitochondrial division in melanoma and diverse disease models and patient samples.

## 2. Materials and Methods

### 2.1. Reagents

Opti-MEM Reduced Serum Media (Gibco, Waltham, MA, USA, Cat. No. 31985-070) was supplemented with 5% FBS, 1% penicillin/streptomycin, 0.01 μg/mL heparin (Sigma-Aldrich, Darmstadt, Germany, Cat. No. H3393), 0.01 μg/μL human FGF-basic (Gibco, Waltham, MA, USA, Cat. No. PHG0026), 0.05 mg/mL dibutyryl cyclic-AMP sodium salt (Sigma-Aldrich, Darmstadt, Germany, Cat. No. DO627), and 0.1 mM 3-isobutyl-1-methylxanthine (Sigma-Aldrich, Darmstadt, Germany, Cat. No. I5789). 2% Tumor Media was prepared with L-15 (Leibovitz) medium (Sigma Aldrich, Darmstadt, Germany, Cat. No. L486) and supplemented with 2.5 μg/mL insulin solution (Sigma Aldrich, Darmstadt, Germany, Cat. No. I0516), 0.187 mg/mL calcium chloride dihydrate (Sigma Aldrich, Darmstadt, Germany, Cat. No. C7902), 1.5 mg/mL sodium bicarbonate (Santa Cruz Biotechnology, Dallas, TX, USA, Cat. No. 203271A), 17.53 g/L MCDB 153 powder (Sigma Aldrich, Darmstadt, Germany, Cat. No. L1518-500ML), 1% penicillin/streptomycin, and 2% FBS. Following preparation, 2% Tumor Media was filtered using a 0.2 micron sterile Nalgene filter unit with polyethersulfone (ThermoFisher-Scientific, Waltham, MA, USA, Cat. No. 567-0020). Dulbecco’s Modification of Eagle’s Medium (DMEM) (Corning, Corning, NY, USA, Cat. No. 10-017-CV) was supplemented with 3% FBS, 1% penicillin/streptomycin, and 1% L-glutamine. The drugs used in this study are GSK1120212 (Selleck Chemicals, Houston, TX, USA, Cat. No. S2673) and PLX4032 (Selleck Chemicals, Houston, TX, USA, Cat. No. S1267). The antibodies utilized for Western blot are total DRP1 (Cell Signaling Technology, Danvers, MA, USA, Cat. No. 8570), DRP1-S616Ⓟ (Cell Signaling Technology, Danvers, MA, USA, Cat. No. 3455S), DRP1-S637Ⓟ (Cell Signaling Technology, Danvers, MA, USA, Cat. No. 4867S), total ERK (Cell Signaling Technology, Danvers, MA, USA, Cat. No. 137F5), ERK1/2Ⓟ (Abcam, Cambridge, UK, Cat. No. AB278538), β-Actin (Abcam, Cambridge, UK, Cat. No. ab8227), TOMM20 (Santa Cruz Biotechnology, Dallas, TX, USA, Cat. No. SC-17764), MFN1 (Santa Cruz Biotechnology, Dallas, TX, USA, Cat. No. SC-166644), MFN2 (Santa Cruz Biotechnology, Dallas, TX, USA, Cat. No. SC-515647), and OPA1 (Invitrogen, Carlsbad, CA, USA, Cat. No. MA5-32786). For microscopy, cells were stained for HSP60 (Cell Signaling Technology, Danvers, MA, USA, Cat. No. 12165) or TOMM20 (Santa Cruz Biotechnology, Dallas, TX, USA, Cat. No. SC-17764), Alexa Fluor^®^ 594-conjugated goat anti-mouse IgG secondary antibody (ThermoFisher-Scientific, Waltham, MA, USA, Cat. No. A11032), and Alexa Fluor^®^ 488-conjugated goat anti-rabbit IgG secondary antibody (ThermoFisher-Scientific, Waltham, MA, USA, Cat. No. A11034). Cells were co-stained with Hoechst 33342 (ThermoFisher-Scientific, Waltham, MA, USA, Cat. No. H3570) to visualize nuclei. For immunohistochemistry, tissues were stained with recombinant 3G11 and BRAF^V600E^ (Cell Signaling Technology, Danvers, MA, USA, Cat. No. 2900S).

### 2.2. Cell Culture

The YUPEET human-derived primary melanoma cell line was cultured in Opti-MEM Reduced Serum Media. The WM35, WM853, WM902, WM983, and WM1152 human-derived primary melanoma lines were cultured in 2% Tumor Media. A375 and SKMEL28 human-derived non-primary (i.e., lymph node and secondary metastasis-derived, respectively) melanoma lines were purchased from ATCC and cultured in DMEM media. All cell lines were cultured at 37 °C in a humidified 5% CO_2_ incubator, passaged every 3–4 days, and regularly tested for mycoplasma contamination.

### 2.3. Immunizations and Hybridoma Generation

Animal experiments were conducted in accordance with the ARRIVE (Animal Research: Reporting of In Vivo Experiments) guidelines and complied with institutional and national regulations for the care and use of laboratory animals. All procedures were approved by the Institutional Animal Care and Use Committee (IACUC) (#IPROTO202200000097). Animals were housed in an AAALAC-accredited facility under controlled environmental conditions (temperature, humidity, and 12 h light/dark cycle) with ad libitum access to food and water. Standard husbandry practices were followed, including routine health monitoring and provision of environmental enrichment. All efforts were made to minimize pain and distress. Blood collection via submandibular vein sampling and surgical procedures, including splenectomy, were performed using approved anesthetic and/or analgesic regimens in accordance with institutional guidelines. Animals were closely monitored throughout the study for signs of pain or distress, including changes in body weight, activity, posture, and grooming behavior. Humane endpoints were predefined, and animals were euthanized if they exhibited signs of significant distress, including sustained weight loss, reduced mobility or inactivity, or failure to groom. The study design adhered to the principles of the 3Rs. Reduction was achieved by using the minimal number of animals necessary to obtain robust results (*n* = 5), and refinement was implemented through appropriate anesthesia, analgesia, and monitoring to minimize discomfort. Replacement alternatives are currently not applicable for hybridoma-based monoclonal antibody generation, which requires in vivo immunization.

For hybridoma generation, five 8-week-old BALB/c female mice were primed subcutaneously (s.c.) with 80 µg (40 µg in each rear flank) of a keyhole limpet hemocyanin (KLH) conjugated DRP1 peptide spanning amino acid residues 607–620, in which Serine 616 was phosphorylated (KLH-Cys-SKPIPIMPA{pSer616}PQKG) (GenScript USA Inc., Piscataway, NJ, USA) in the presence of complete Freund’s adjuvant (CFA). Four weeks after priming, a total of two more boosts commenced in the same manner in the presence of incomplete Freund’s adjuvant. After immunizations, blood was collected from the submandibular vein before each boost to monitor the titer of serum antibodies by enzyme-linked fluorescent immunosorbent assay (ELISA).

For the ELISA, plates (ThermoFisher Scientific, Waltham, MA, USA, Cat. No. 2205) were coated with 100 µL of 10 µg/mL peptide in 1× PBS, followed by blocking with 1% BSA/PBS and washing (3×) using a BioTek 405 microplate washer (Agilent Technologies, Santa Clara, CA, USA). Serum was diluted in 1% BSA/PBS and added to the coated well. After 3× washes, goat anti-mouse IgG-HRP (1:4000 in 1% BSA/PBS) (Jackson ImmunoResearch Labs, West Grove, PA, USA; Cat. No. 115-035-003) was added and positive wells were detected with ABTS substrate solution (Roche, Sommerville, CA, USA, Cat. No. 11684302001). All incubation steps were performed at 25 °C for 1 h. Unimmunized sera were also added as a negative control. The mouse with the highest titer at the 1:10,000 dilution that was also >2× the negative control was selected for hybridoma fusion and received 2 final boosts of 150 µg BSA-conjugated phosphorylated-DRP1 peptide s.c. (50 µg in 3 sites) over 2 days. A final bleeding (“fusion sera”) and terminal splenectomy were performed 3 days after the last final boost. The spleen was processed to a single-cell suspension and fused in the presence of ClonaCell HY-PEG (Stemcell Technologies, Vancouver, BC, Canada; Cat. No. 03806) with Sp2/0 hetero-myeloma fusion partners. Individual hybridoma B cell clones were grown under selection on soft agar containing hypoxanthine-aminopterin-thymidine (HAT), and expanded colonies were picked ten days later into 96-well tissue culture plates using a robotic ClonaCell Easy Pick instrument (Hamilton/Stem Cell Technology, Vancouver, BC, Canada) in ClonaCell HY-Medium E (Stemcell Technologies; Vancouver, BC, Canada; Cat. No. 03805). Clones were expanded, and the supernatant was used to screen for DRP1 detections.

### 2.4. Dot Blot Analyses

Indicated peptides were solubilized in 1× PBS, 10 µL of 10 µg/mL of peptide was manually spotted onto nitrocellulose membranes, and the membranes were dried. Membranes were rehydrated in 1× PBST and blocked in 4% nonfat dry milk in 1× PBST. Membranes were incubated with either the indicated anti-serum or commercially available DRP1 and DRP1-S616Ⓟ antibodies diluted in blocking buffer (1:1000, 4% milk in 1× PBST) at 4 °C for 14 to 16 h. After incubation, blots were washed with 1× PBST and visualized using enhanced chemiluminescence detection (Millipore, Burlington, MA, USA, Cat. No. WBLUF0100).

### 2.5. Cloning and Expression of the 3G11 Antibody

Sequencing of the 3G11 antibody was performed using SMARTer 5′ RACE technology (Takara Bio, MoutainView, CA, USA, Cat. No. 634858) adapted for immunoglobulins to amplify the variable genes from the heavy and kappa chains from the 3G11 hybridoma. Briefly, RNA was extracted from the hybridoma using a RNeasy Mini Kit (Qiagen, Hilden, Germany, Cat. No. 74104), followed by first-strand cDNA synthesis using constant gene-specific 3′ primers (GSP1) based on mouse IgG/mouse kappa constant isotypes and incubation with the SMARTer II A Oligonucleotide and SMARTscribe reverse transcriptase (Takara, MoutainView, CA, USA).

GSP1 Primers (5′–3′):

mG1-AGAGGTCAGACTGCAGGACA, mG2a- CTTGTCCACTTTGGTGCTGC,
mG2b-GACAGTCACTGAGCTGCTCA, mG2b-GACAGTCACTGAGCTGCTCA,
and mcK-CCAACTGTTCAGGACGCCAT.

PCR amplification of the first-strand cDNA product was then performed using SeqAmp DNA Polymerase (Takara, MoutainView, CA, USA, Cat. No. 638504) with a nested 3′ primer (GSP2 Primer) to the constant genes and a 5′ universal primer (kit provided) based on universal primer sites added to the 5′ end during cDNA generation.

GSP2 Primers (5′–3′):

mG1-CCCAGGGTCACCATGGAGTT, mG2a-GGTCACTGGCTCAGGGAAAT,
mG2b-CTTGACCAGGCATCCCAGAG, mG3-GACAGGGCTCCATAGTTCCATT,
and mCk-CTGAGGCACCTCCAGATGTTAAC.

Purified PCR products were submitted for Sanger sequencing using 3′ constant gene primers (GeneWiz, South Plainfield, NJ, USA). Sequence results were BLASTed against the IMGT mouse databank of germline genes using V-Quest (https://www.imgt.org/, accessed on 8 January 2026). Complete 3G11 heavy and kappa chain variable region cDNAs were then synthesized with an Il-2 signal sequence and cloned into a pcDNA-3.4 mammalian expression vector in-frame on the 3′ end to a vector-encoded mouse IgG2a constant region (heavy chain) or a mouse kappa constant region (light chain) (GenScript USA Inc., Piscataway, NJ, USA). Cloned vectors representing full heavy and kappa chains were then transfected in a 1:1 ratio into HEK Expi293 suspension cells using ExpiFectamine transfection reagent following manufacturer instructions (ThermoFisher, Waltham, MA, USA, Cat. No. A14524). Supernatant was collected 5 days after initial transfection, filtered (0.45 micron), and subjected to quantitation/verification by bio-layer interferometry (BLI) and purification.

### 2.6. Quantitation and Verification of 3G11 Supernatant 

For BLI analysis, 3G11 supernatant was quantitated and verified on an Octet Red96 (ForteBio, Sartorius, Goettiengen, Germany). Optically capable biosensors conjugated with Protein A were dipped into supernatants containing the 3G11 mAb. Supernatants were measured undiluted and diluted 1:10 in conditioned media and compared to an isotype standard (mIgG2a), diluted in conditioned media in a 1:2 dilution series ranging from 100 µg/mL to 1.56 µg/mL. Standard curves were analyzed in the ForteBio Data Analysis Software v.10 (ForteBio, Goettiengen, Germany) using a 5 parameter logistics (5PL) dose response curve fitting model on the initial binding slopes. MAb concentrations are then calculated from the standard curve. Diluted samples were compared to undiluted samples after application of the respective dilution factors. Total concentrations were averaged together from the diluted and undiluted samples.

### 2.7. 3G11 Antibody Purification

The supernatant was purified on an ÄKTA pure FPLC system using a Protein-A affinity column (HiTrap-1 mL, GE/Cytiva, Uppsala, Sweden, #17-0404-01) with UV monitored extraction. A single UV peak fraction was collected, representative of the antibody, dialyzed against PBS and quantitated by both bicinchoninic assay (BCA) (Pierce Biotechnology, Rockford, IL, USA, Cat. No. 23225) and absorption at OD 280 nm. 3G11 yielded ~4–10 μg/mL across ~300 mL of culture over three production runs, resulting in ~2.1 mg total purified antibody.

### 2.8. In Vitro Kinase Assay and Analysis

Recombinant, GST-tagged, full-length, human DRP1 protein (10 ng/μL) [19] was incubated with recombinant ERK1 kinase (2 ng/μL; Sigma, Darmstadt, Germany, Cat. No. 14-439-M) in kinase buffer (50 mM Tris-HCl pH 7.4, 30 mM NaCl, 15 mM MgCl_2_, 200 μg/mL BSA, 2 mM DTT, and 1 mM ATP) at 37 °C for 30 min. The reaction was terminated by the addition of 4× SDS loading buffer (0.2 M Tris-HCl, 0.4 M 2-mercaptoethanol, 277 mM SDS, 6 mM Bromophenol Blue, 4.3 M Glycerol). DRP1 (100 ng/lane) was subjected to SDS-PAGE, followed by standard Western blotting. The nitrocellulose membrane was blocked in 5% dried nonfat milk in 1× TBST and incubated with primary antibodies (1:1000; 5% milk in 1× TBST) at 4 °C for 14 to 16 h. The membrane was then washed 3 times with 1× TBST, incubated with the secondary antibody (1:2500; 5% milk in 1× TBST) at 25 °C for 1 h, washed 3 times with 1× TBST, and detected using enhanced chemiluminescence (Millipore, Burlington, MA, USA Cat. No. WBLUF0100).

### 2.9. Lentiviral Short Hairpin RNA (shRNA) Generation

pLKO control (SHC007, TRC2), sh1-DNM1L (TRCN0000001097), and sh2-DNM1L (TRCN0000001099) lentiviral vectors were obtained from Sigma Mission shRNA. Lentiviral particles were produced in HEK293T cells. In brief, HEK293T cells were seeded in three 10 cm dishes at a density of 4 × 10^6^ cells per dish. The following day, cells were transfected using Lipofectamine 2000 (Thermo Fisher Scientific, Waltham, MA, USA, Cat No. 11668019) in Opti-MEM (Gibco, Waltham, MA, USA, Cat. No.31985-070). For each viral construct, two transfection mixtures were prepared. The first contained 12 µL Lipofectamine 2000 diluted in 250 µL Opti-MEM. The second contained 5 µg of the transfer vector, together with 2 µg of each of the packaging plasmids psPAX2 (Addgene, Watertown, MA, USA, Cat. No. 12260) and pMD2.G (Addgene, Watertown, MA, USA, Cat. No. 12259), diluted in 250 µL Opti-MEM. Each mixture was incubated separately for 5 min at room temperature, then combined and incubated for an additional 15 min to allow complex formation. The transfection mixture was added dropwise to the HEK293T cells. Cells were incubated at 37 °C for 16 h, after which the transfection medium was replaced with fresh DMEM. Viral supernatants were collected at 24 and 48 h after medium change, for a total collection volume of 20 mL per construct. The collected supernatants were passed through a 0.45 micron filter to remove cellular debris. Lentiviral particles were then concentrated using Lenti-X Concentrator (Takara, MoutainView, CA, USA, Cat. No. 631232) according to the manufacturer. The final volume of concentrated lentivirus was 200 µL.

For transduction, YUPEET cells were seeded at 0.2 × 10^6^ cells per 6 cm dish for RNA analysis. Cells were transduced with concentrated lentivirus at 20 µL per 10 cm dish or 10 µL per 6 cm dish and incubated at 37 °C for 48 h. The medium was then replaced with fresh medium containing 2 µg/mL of puromycin (Gibco, Waltham, MA, USA, A11138-03) for selection. After 48 h of puromycin selection, cells were maintained in 1 µg/mL puromycin for 7 days, and harvested for SDS-PAGE/Western blot or RNA extraction.

### 2.10. Reverse Transcription Polymerase Chain Reaction

Total RNA was extracted from cell pellets with the RNeasy mini kit (Qiagen, Hilden, Germany, Cat. No. 74104), following the manufacturer’s instructions. RNA was quantified using a Nanodrop One Spectrophotometer. 2 μg of RNA was used to synthesize cDNA using the RNA to cDNA EcoDryTM Premix Double Primed 28 (Takara Bio, MoutainView, CA, USA, Cat. No. 639548). Reaction tubes were placed in the thermocycler and subjected to 42 °C for 1 h and 70 °C for 10 min, followed by an indefinite hold at 4 °C until they were moved to storage at −20 °C. Forward and reverse primers for genes of interest (see Table 1 below) were combined with *Power* SYBR Green Master Mix (Applied Biosystems, Carlsbad, CA, USA, Cat. No. 437659), and gene expression was analyzed using a ViiA7 Real-Time PCR system. The expression of relevant genes was normalized to *18S.*

### 2.11. Whole Cell Lysate Isolation and Analysis

One 10 cm dish of cells at 80% confluency was used per condition. Cells were treated with DMSO, GSK1120212 (50 nM), or PLX4032 (1 μM) for 6 h. Cells were trypsinized, pelleted by centrifugation at 800× *g* for 10 min, washed with pre-chilled 1× PBS, and pelleted again using the same parameters. The cell pellets were lysed by resuspending in 1× RIPA buffer supplemented with protease inhibitors (ThermoFisher-Scientific, Waltham, MA, USA, HALT Tablet, Cat. No. 87786) and phosphatase inhibitors (ApexBio, Houston, TX, USA, Cat. No. k1015b). The suspension was incubated on ice for 10 min and then centrifuged at 21,000× *g* at 4 °C for 10 min. Protein concentrations were determined using a BCA assay. Cell lysates were adjusted with 1× RIPA buffer to achieve the same protein concentration across samples. 4× SDS loading buffer(0.2 M Tris-HCl, 0.4 M 2-mercaptoethanol, 277 mM SDS, 6 mM Bromophenol Blue, 4.3 M Glycerol) was added to each sample and diluted to a final 1× concentration, Samples were then boiled at 95 °C for 5 min. 75 μg of cell lysate per lane was subjected to SDS-PAGE followed by transferring to a nitrocellulose membrane. The nitrocellulose membrane was blocked in 5% milk/1× TBST and then incubated with primary antibodies (1:1000, 5% milk in 1× TBST) at 4 °C for 14–16 h. The membrane was then incubated with the secondary antibody (1:2500, 5% milk in 1× TBST) at 25 °C for 1 h before enhanced chemiluminescence detection (Millipore, Burlington, MA, USA Cat. No. WBLUF0100).

### 2.12. Heavy Membrane Isolation and Analysis

At least two 15 cm dishes of cells at 90–95% confluency were used per condition for heavy membrane (i.e., mitochondrial fractions) isolations. Cells were treated with DMSO, GSK1120212 (50 nM), or PLX4032 (1 μM) for 6 h. The cells were harvested by trypsinization and pelleted by centrifugation at 800× *g* at 4 °C for 10 min. The cell pellets were washed with 1 mL of pre-chilled 1× PBS, pelleted, washed with 1 mL of pre-chilled trehalose isolation buffer (TIB) [300 mM Trehalose, 10 mM HEPES-KOH (pH 7), 10 mM KCl, 1 mM EDTA, 1 mM EGTA], and pelleted by centrifugation at 800× *g* for 5 min. The cell pellets were resuspended with TIB (supplemented with 1× protease inhibitors, ThermoFisher-Scientific, Waltham, MA, USA, HALT Tablet, Cat. No. 87786) at a 1:1 volume and incubated on ice for 30 min to allow for cell swelling. The suspension was then transferred into a pre-chilled 2 mL Potter-Elvehjem dounce and homogenized with fifty strokes to reach 70–80% cell lysis. The homogenate was then transferred to a 1.5 mL Eppendorf tube and centrifuged at 1000× *g* at 4 °C for 5 min. The supernatant was collected and centrifuged again under the same conditions to ensure that no unlysed cells or nuclei were present. The resulting supernatants were centrifuged at 10,000× *g* at 4 °C for 10 min, and the pellets were collected as the heavy membrane fraction. Sample yield was determined using the Pierce BCA Assay Kit, cell lysates were adjusted with 1× RIPA buffer to achieve the same protein concentration across all samples, and 4× SDS loading buffer was added. 50 μg of cell lysate was loaded per lane, and analyzed by SDS-PAGE and Western blotting.

### 2.13. Lambda Phosphatase Treatment

YUPEET RIPA lysates were prepared at an initial protein concentration of 16.99 μg/μL. Lysates (100 μg) were brought to a final volume of 40 μL with molecular biology-grade water. Reactions were supplemented with 5 μL of 10× NEBuffer for Protein MetalloPhosphatases (New England Biolabs, Ipswich, MA, USA, Cat. No. B0761) and 5 μL of 10 mM MnCl_2_ (New England Biolabs, Ipswich, MA, USA, Cat. No. B1761). Lambda protein phosphatase (New England Biolabs, Ipswich, MA, USA, Cat. No. P0753S) was added (2 μL) for a total of 800 U/reaction. For untreated controls, 2 μL of molecular biology-grade water was added in place of the enzyme. Samples were mixed gently and briefly centrifuged. Reactions were incubated at 30 °C for 30 min. Following incubation, Laemmli buffer was added to each sample, which were then boiled at 95 °C for 5 min and briefly centrifuged prior to downstream analysis by SDS-PAGE and Western blotting.

### 2.14. Immunofluorescence (IF)

A375 and SKMEL28 cells were seeded on 1.5 mm coverslips (Electron Microscopy Science, Hatfield, PA, USA, Cat. No. 72230-01) and allowed to adhere for 24 h. The next day, cells were treated with GSK1120212 (50 nM) for 16 h. After treatment, cells were washed 3 times with 1× PBS and fixed in 4% formaldehyde for 15 min. The cells were then washed another 3 times in 1× PBS and then permeabilized using 0.3% Triton X-100 at 25 °C for 12 min. Cells were washed again 3 times with 1× PBS and subsequently incubated in the blocking buffer (50% 1× PBS, 4% BSA, and 10% goat serum) at 25 °C for 2 h. Coverslips were incubated with primary antibodies (3G11, 1:3200; TOMM20, 1:400; HSP60:, 1:400) diluted in the blocking buffer at 4 °C for 14–16 h. Secondary antibodies Alexa Fluor^®^ 594 (1:250) and Alexa Fluor^®^ 488 (1:250) were diluted in the blocking buffer and incubated at 25 °C for 1 h. Samples were treated with Hoechst 33342 (1:5000), mounted with ProLong Glass (ThermoFisher, Waltham, MA, USA, Cat. No. P36980) and cured at 4 °C overnight.

Images were captured at the Mount Sinai Tisch Cancer Center Microscopy and Advanced Bioimaging Shared Resource. Images were acquired with LAS X software (Leica Application Suite X 4.3.0.24308) on Leica Stellaris 8 (Leica Microsystems GmbH, Wetzlar, Germany) confocal microscope. Imaging was performed using a 63×/1.3 HC PlanApo oil immersion lens (Leica Microsystems GmbH, Wetzlar, Germany), the frame size was set to 2048 × 2048 pixels (X/Y), and images were acquired at 8 bits. The acquisition was performed at frame scan mode, using 1 frame averaging and 2 line accumulation. Fluorophores were excited with a 440 nm laser derived from an 80 MHz pulsed White Light Laser (Leica Microsystems GmbH, Wetzlar, Germany). The fluorophore emission was collected with Hybrid Detectors (HyDS, Leica Microsystems GmbH, Wetzlar, Germany) with a spectral window of 445–650 nm. The laser power and gain were set to provide the best signal-to-noise ratio for each chromophore while utilizing the full dynamic range of the detector and avoiding detector saturation.

Image analysis and mitochondrial quantification were performed using ImageJ’s plugins MitochondrialAnalyzer-V2.3.1 and DeconvolutionLab2_2.0.0. All images were pre-processed manually, which included masking the image to focus on a single cell (if there are multiple cells in the frame), performing a background subtraction, deconvolution (if not done through microscopy software while imaging), and image thresholding. Background subtraction was performed with a 4-pixel rolling-ball radius to minimize the background noise while still maintaining the integrity of the mitochondrial structure. Deconvolution was performed through the DeconvolutionLab2 plugin and was optimized for these images with respect to resolution, morphology structure, image size, and overall image quality. A Gaussian PSF with a Richardson-Lucy algorithm (15 iterations) was used to develop the deconvolved image. Once deconvolved, all images were auto-thresholded using Moments from ImageJ (1.54r). Moments creates a binary based on the pixel intensity in the original image histogram and is a very consistent thresholding option. Mitochondrial Analyzer2D Analysis provides per-mitochondrion and summary data for each cell, using the binary image to identify total count, branch count, branch length, and additional metrics. For the purposes of elongation and fragmentation, the area-to-perimeter and perimeter-to-area ratios were calculated to better illustrate the level of fragmentation between groups. A power test revealed that 21 cells were necessary to establish statistical significance at a 5% significance level, with a *t*-test used to evaluate *p*-values.

### 2.15. Immunohistochemistry (IHC)

De-identified formalin-fixed paraffin-embedded (FFPE) human skin sample biopsy sections (5 micron thickness) were obtained from the Mount Sinai Tisch Cancer Center Biorepository and Pathology Shared Resource. A Leica BOND RX automated system for immunohistochemistry was utilized to stain tissue samples using a Bond Polymer Refine Red Detection Kit, which contains polymeric alkaline phosphatase (AP) conjugate as a secondary reagent to detect primary antibodies (Leica Biosystems, Nussloch, Germany, Cat. No. DS9390). Primary antibodies (were diluted in BOND Primary Antibody Diluent (Leica Biosystems, Cat. No. AR9352) as indicated: 3G11 (1:1000) and BRAF^V600E^ (1:150). Bond Epitope Retrieval (ER) Solution 2 (Leica Biosystems, Cat. No. AR9640) was used for 20 min; the slides were then mounted with DAKO Glycergel mounting medium (Agilent Technologies, Santa Clara, CA, USA, Cat. No. C0563) and imaged. Slides were scored by a binary measure (0 or 1), which represents the absence/presence of stain, and a subset of samples was confirmed by pathologists within the Mount Sinai Division of Dermatopathology. Scoring was analyzed via Fisher’s Exact Test and a Chi-Squared Test with Yates correction. Statistics were performed with GraphPad Prism software (11.0.0). The Program for the Protection of Human Subjects office determined that the above study is exempt from human research (HS#13-00606) as defined by DHHS regulations (45CFR46.101(b)(4)).

## 3. Results

### 3.1. Antibody Development Workflow and Screening to Identify 3G11 Anti-Sera

**Figure 1:**
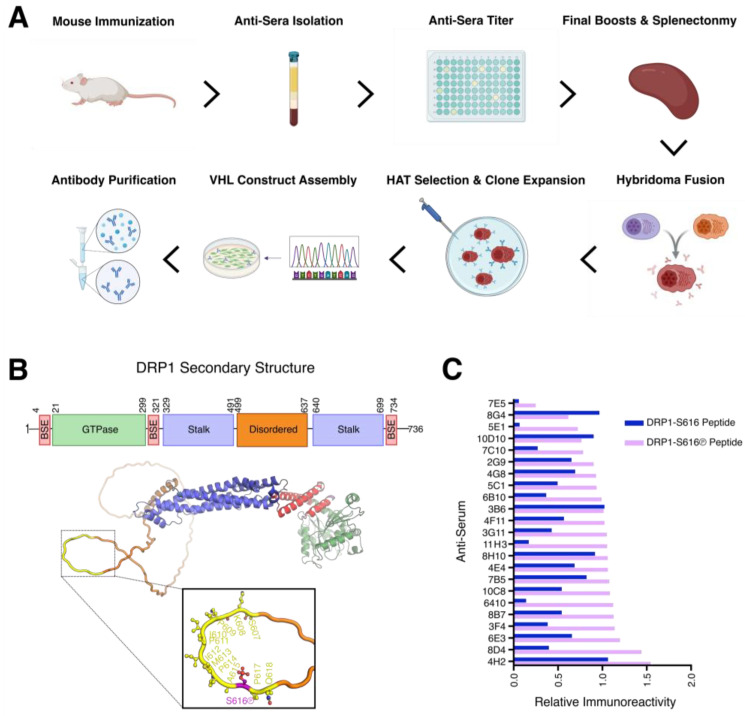
Antibody development workflow, DRP1 domains, and anti-sera screening. (**A**) Schematic representation of the workflow to generate the monoclonal recombinant 3G11 antibody, including: immunization, anti-sera screening, hybridoma development, and identification. HAT and VH/L are Hypoxanthine/Aminopterin/Thymidine and Variable Heavy/Light chain, respectively. (**B**) Predicted three-dimensional structure of human DRP1 (AlphaFold V2, AF-O00429-v4), rendered using PyMOL (3.1.6, Schrodinger LLC.), with structural domains color-coded based on domain definitions (Rochon et al., 2024) [20]. The epitope recognized by the 3G11 antibody is highlighted in yellow, and DRP1-S616Ⓟ is shown in purple. (**C**) ELISA results comparing the indicated anti-sera for reactivity to the DRP1 serine 616 non-phosphorylated and phosphorylated peptides.

To develop a recombinant monoclonal antibody that specifically detects DRP1-S616Ⓟ, we designed an immunization strategy using a keyhole limpet hemocyanin (KLH)-conjugated peptide corresponding to human DRP1 amino acid residues 607–620, with serine 616 phosphorylated. This phospho-peptide served as the immunogen for mouse immunizations, and subsequent hybridomas were generated by fusing splenic B cells from immunized mice with Sp2/0 hetero-myeloma cells [20]. Once the hybridomas were screened, the optimal VH/L regions were sequenced, cloned, and purified; the workflow is summarized (Figure 1A). The immunogen was selected by integrating sequence and structural context from an AlphaFold-predicted model of full-length human DRP1, as depicted in (Figure 1B). To identify anti-sera capable of specifically recognizing the serine 616 phosphorylated epitope, we performed an ELISA assay comparing interactions between the DRP1-S616 and DRP1-S616Ⓟ peptides (Figure 1C). Anti-sera that exhibited strong reactivity were further evaluated using dot blot analysis to confirm selectivity for the phosphorylated DRP1 sequence (Figure S1A–C). Based on the specificity for the DRP1-S616Ⓟ peptide by ELISA and dot blot, along with anti-sera freeze/thaw stability and protein quantifications, we selected five mice: 2G9, 3G11, 5E1, 10C8, and 10D10 for hybridoma generation and further validation.

**Figure 2:**
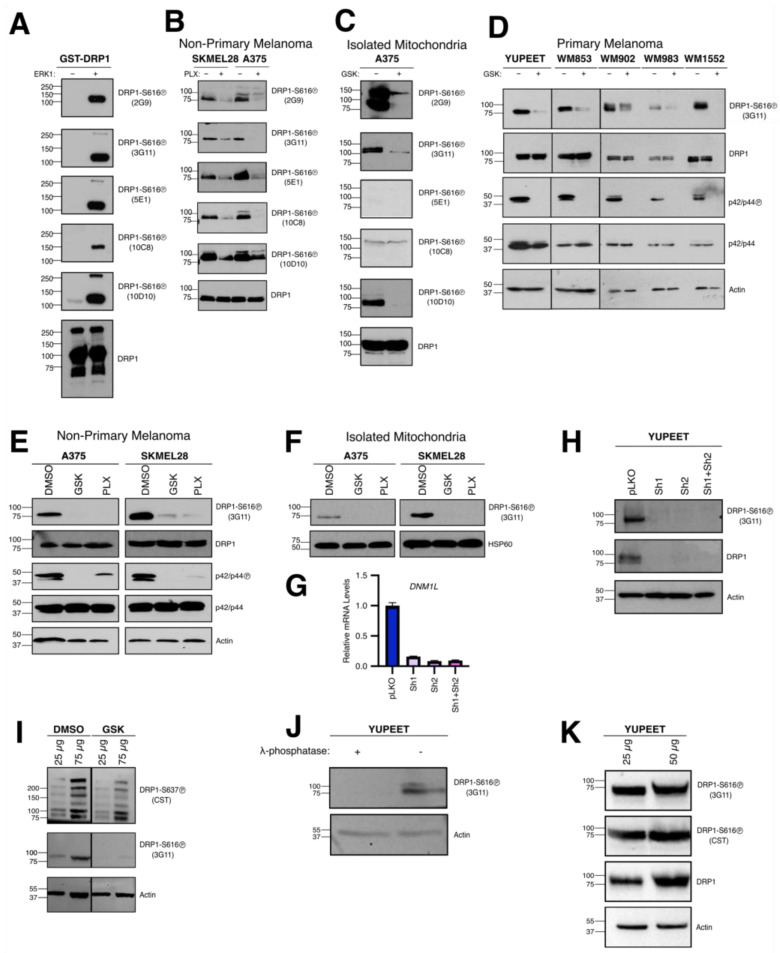
Hybridoma screenings and recombinant 3G11 antibody evaluation. (**A**) Hybridomas generated from the top five anti-sera were evaluated for specificity using Western blot analysis of full-length recombinant GST-DRP1 ± ERK1 treatment. 100 ng of recombinant GST-DRP1 was loaded per lane. Total DRP1 was evaluated for equal protein loading. (**B**,**C**) The hybridomas were screened using Western blot analysis of whole cell lysates (100 µg/lane; (**B**)) or heavy membrane fractions (25 µg/lane; (**C**)) isolated from SKMEL28 and A375 cells treated with GSK1120212 (50 nM; GSK), PLX4032 (1 µM; PLX), or DMSO for 6 h. Total DRP1 was evaluated for equal protein loading. (**D**) Primary melanoma lines were treated with GSK1120212 (50 nM) or PLX4032 (1 µM) for 6 h. Whole cell lysates were Western blotted for the indicated proteins. Recombinant 3G11 was evaluated for DRP1-S616Ⓟ detection. Actin was probed for equal protein loading. (**E**) A375 and SKMEL28 were treated with GSK1120212 (50 nM) or PLX4032 (1 µM) for 6 h and evaluated as in *D*. Recombinant 3G11 was evaluated for DRP1-S616Ⓟ detection. Actin was probed for equal protein loading. (**F**) Heavy membrane fractions from the same treatments in D were Western blotted for the indicated proteins. Recombinant 3G11 was evaluated for DRP1-S616Ⓟ. HSP60 was probed for equal protein loading. Molecular weight standards for all Western blots are indicated in kilodaltons (kDa). (**G**,**H**) YUPEET cells expressing two independent shRNAs targeting *DNM1L* (sh1, sh2), or their combination, were analyzed by RT-qPCR and Western blot to determine *DNM1L* knockdown and loss of detection by recombinant 3G11. (**I**) YUPEET cells were treated with GSK1120212 (50 nM) or DMSO for 6 h, and whole cell lysates were evaluated by SDS-PAGE and Western blot for the indicated proteins to assess the specificity of recombinant 3G11 for DRP1-S616 phosphorylation relative to S637. Actin was used as a loading control. (**J**) YUPEET cell lysates were treated with λ-phosphatase for 30 min prior to SDS-PAGE and Western blot analysis. Actin was used as a loading control. (**K**) Untreated whole cell lysates (25 and 50 μg) from YUPEET cells were analyzed by SDS-PAGE and Western blot to compare the sensitivity of recombinant 3G11 and the Cell Signaling Technology (CST) antibody. Total DRP1 and actin were used as loading controls. Data are representative of three independent experiments.

We next determined which of these hybridomas distinguished between unphosphorylated and phosphorylated full-length, recombinant, human DRP1. First, we performed an in vitro kinase assay to specifically phosphorylate DRP1 at serine 616 using recombinant ERK1, as described [9,10]. DRP1-S616 and DRP1-S616Ⓟ were subjected to SDS-PAGE and Western blot, and the 5 hybridomas were analyzed (Figure 2A).

Next, we assessed the ability of these hybridomas to detect endogenous DRP1-S616Ⓟ in whole cell lysates isolated from BRAF^V600E^-positive human malignant melanoma cell lines (i.e., A375 and SKMEL28) (Figure 2B). To functionally validate the clones’ specificities, we also treated these cells with PLX4032 (or DMSO), a targeted therapy against BRAF^V600E^, which inhibits downstream endogenous ERK1/2 from phosphorylating DRP1 at serine 616 [9]. Western blot analysis was performed to compare DRP1-S616Ⓟ levels in DMSO versus PLX4032-treated cells. Among the five hybridomas, 3G11 demonstrated the strongest and most specific detection of DRP1-S616Ⓟ in DMSO-treated cells, with a potent reduction in DRP1-S616Ⓟ following PLX4032 exposure, indicating that 3G11 effectively detects endogenous DRP1-S616Ⓟ (Figure 2B).

As DRP1-S616Ⓟ accumulates at the OMM [7], we subsequently evaluated which hybridoma could detect activated DRP1-S616Ⓟ localized to endogenous mitochondria. A375 cells were treated with GSK1120212 (or DMSO), a MEK inhibitor that suppresses ERK1/2, prior to isolating the mitochondrial fraction. SDS-PAGE and Western blot analysis of isolated mitochondrial proteins revealed that the 3G11 anti-serum potently detected the presence of DRP1-S616Ⓟ in DMSO-treated samples with minimal background; furthermore, there was an appreciable decrease in DRP1-S616Ⓟ detection following GSK1120212 treatment (Figure 2C).

### 3.2. The Recombinant 3G11 Antibody Specifically Detects DRP1-S616 Phosphorylation

Through our screening process, we identified 3G11 as the optimal clone for detecting DRP1-S616Ⓟ based on its peptide specificity in ELISA and dot blot studies, and Western blot analyses using whole cell lysates and isolated mitochondrial fractions (Figure 1 and Figure 2). Therefore, we sequenced and cloned the 3G11 VH/L regions and generated a purified recombinant monoclonal version of the 3G11 antibody. Using the purified recombinant 3G11 antibody, we proceeded to examine its ability to detect DRP1-S616Ⓟ in several melanoma models before and after oncogenic MAPK pathway inhibition.

To broaden our approach, we expanded our analyses to include a panel of primary melanoma cell lines harboring the BRAF^V600E^ mutation (i.e., YUPEET, WM853, WM902, WM983, and WM1552) treated with GSK1120212 (or DMSO). SDS-PAGE and Western blot analyses of whole cell lysates from these cell models revealed that ERK1/2 inhibition led to a significant reduction in 3G11 detection of DRP1-S616Ⓟ levels compared to DMSO-treated cells (Figure 2D). Inhibition of MAPK signaling was also confirmed by a corresponding reduction in p42/p44 (ERK1/2) phosphorylation in all cell lines to ensure GSK1120212 treatments were effective. Together, these data indicate a selective effect of oncogenic MAPK pathway inhibition on DRP1-S616Ⓟ.

Next, to further validate recombinant 3G11’s ability to detect DRP1-S616Ⓟ across different melanoma models, we extended our analysis to the non-primary BRAF^V600E^ melanoma cell lines, A375 and SKMEL28. As in Figure 2B, we treated the cell lines with either GSK1120212 or PLX4032 and performed Western blot analysis of whole cell lysates. In both cell lines, DRP1-S616Ⓟ levels decreased upon treatment with either inhibitor, while total DRP1 expression remained unchanged, confirming that recombinant 3G11 specifically detects DRP1-S616Ⓟ (Figure 2E).

We further evaluated whether recombinant 3G11 detected DRP1-S616Ⓟ in isolated mitochondrial fractions. Mitochondria were isolated from A375 and SKMEL28 cells treated with either GSK1120212 or PLX4032, and SDS-PAGE/Western blot analysis was performed. In both cell lines, DRP1-S616Ⓟ levels decreased in the mitochondrial fractions following oncogenic MAPK inhibition, while total mitochondrial DRP1 remained unchanged (Figure 2F).

To further substantiate the specificity of 3G11 for DRP1, we silenced *DNM1L* expression in YUPEET cells using two independent shRNA constructs. Knockdown efficiency was confirmed by RT–qPCR and immunoblotting, demonstrating a pronounced reduction in both total DRP1 protein levels and DRP1-S616Ⓟ signal (Figure 2G,H). These findings indicate that 3G11 immunoreactivity is dependent on *DNM1L* expression, thereby reinforcing its target specificity.

We next assessed whether 3G11 selectively detects phosphorylation of DRP1 at serine 616 and not serine 637, the major inhibitory site for DRP1 [21,22,23]. YUPEET cells were treated with GSK1120212 for 6 h to inhibit MAPK signaling, followed by Western blot analysis of DRP1-S616Ⓟ and DRP1-S637Ⓟ status. Inhibition of MAPK signaling resulted in a reduction in the Serine 616 phosphorylation signal detected by 3G11, while Serine 637 phosphorylation remained unchanged (Figure 2I), indicating site-specific recognition.

To biochemically validate phospho-epitope specificity, whole-cell lysates derived from YUPEET cells were subjected to enzymatic dephosphorylation using λ-phosphatase prior to SDS-PAGE and Western blot analysis. This treatment, performed under conditions that preserve protein integrity while catalytically removing phosphate moieties from serine, threonine, and tyrosine residues, resulted in complete abrogation of the 3G11 immunoreactive signal relative to untreated control lysates (Figure 2J). In contrast, total DRP1 protein levels remained unchanged, excluding the possibility of nonspecific protein degradation or loss during sample processing. The selective elimination of signal following phosphatase treatment demonstrates that 3G11 recognition is strictly dependent on the presence of a phosphorylated epitope, thereby providing strong biochemical evidence for its phospho-specificity.

Finally, to benchmark the performance of recombinant 3G11 against a commercially available antibody, we compared the sensitivity of 3G11 to the Cell Signaling Technology (CST) DRP1-S616Ⓟ antibody. Untreated whole cell lysates were prepared, two masses of total protein were loaded, and analyzed by SDS-PAGE and Western blot. Across both input levels, recombinant 3G11 demonstrated comparable detection of DRP1-S616Ⓟ relative to the commercial antibody; total DRP1 and actin also confirmed loading (Figure 2K). These findings further support the sensitivity and reliability of recombinant 3G11 for detecting DRP1-S616 phosphorylation.

Together, these data demonstrate that recombinant 3G11 selectively and specifically detects serine 616 phosphorylation of DRP1 and is suitable for assessing its regulation across multiple experimental systems.

### 3.3. Recombinant 3G11 Detects Oncogenic MAPK-Regulated DRP1-S616 Phosphorylation and Mitochondrial Co-Localization

**Figure 3:**
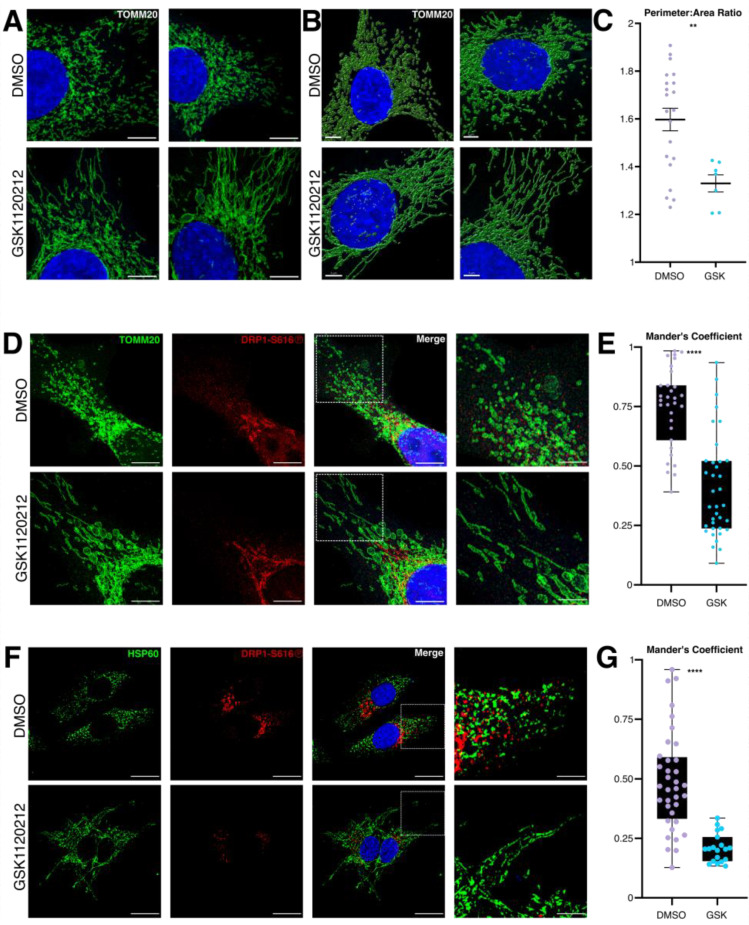
Oncogenic MAPK-regulated DRP1-S616Ⓟ expression is detected by recombinant 3G11. (**A**) SKMEL28 cells were treated with GSK1120212 (50 nM) for 16 h, fixed, and stained for TOMM20 (green; mitochondria) and Hoechst 33342 (blue; nuclei). Scale bars = 10 microns. (**B**) SKMEL28 cells were studied as in (**A**). Mitochondrial networks are shown as maximum intensity projections of super-resolution Leica LIGHTNING deconvolved confocal z-stacks. Imaris surface features were used to generate 3D projections to visualize changes in mitochondrial network architecture. Scales bars = 5 microns. (**C**) Mitochondrial elongation was quantified using the Perimeter: Area Ratio. (**D**,**E**) SKMEL28 cells were treated with GSK1120212 (50 nM) for 16 h, fixed, and stained for TOMM20 (green), DRP1-S616Ⓟ (red), and Hoechst 33342 (blue). Red channel intensity was quantified to assess DRP1-S616Ⓟ signal intensity using Mander’s coefficient. Scale bars = 10 microns. Zoomed network images (white dashed box) = 50 micron scale bars. (**F**,**G**) A375 cells were analyzed as panels (**D**,**E**). All experiments were performed at least twice, with statistical significance determined from a minimum of two independent experiments using a one-way ANOVA test. ** refers to a *p* value ≤ 0.01 and **** refers to a *p* value ≤ 0.0001.

To evaluate the utility of recombinant 3G11 in immunofluorescence (IF) based applications, we performed immunostaining using the BRAF^V600E^ non-primary melanoma cells (n.b., A375 and SKMEL28 display more organized mitochondrial networks compared to the primary melanoma lines, which eases imaging analyses). SKMEL28 cells were treated with DMSO or GSK1120212 (50 nM, 16 h), fixed, and immuno-stained using recombinant 3G11 to detect DRP1-S616Ⓟ, TOMM20 (OMM marker), and Hoechst 33342 (nuclear stain). First, we confirmed that GSK1120212 treatment inhibited mitochondrial division and resulted in elongated mitochondrial networks. Both standard 2D images and 3D projections of GSK1120212-treated SKMEL28 cells revealed elongated mitochondrial networks, confirming effective pathway modulation (Figure 3A,B). This effect was corroborated with the quantification of the perimeter-to-area ratio (Figure 3C). Next, we assessed the effects of oncogenic MAPK inhibition on DRP1-S616Ⓟ signal and its co-localization with mitochondria. In DMSO-treated cells, DRP1-S616Ⓟ was prominently detected and exhibited co-localization with mitochondria (Figure 3D). After GSK1120212 treatment, DRP1-S616Ⓟ expression was reduced, with a corresponding decrease in mitochondrial co-localization, as quantified using Mander’s coefficient (Figure 3D,E). These findings were recapitulated in A375 cells, where GSK1120212 treatment similarly reduced DRP1-S616Ⓟ signal detected by 3G11 (Figure 3F,G). Together, these results support the utility of recombinant 3G11 for detecting phosphorylation-dependent changes in DRP1 localization by immunofluorescence, demonstrating that 3G11 sensitively reports MAPK-regulated modulation of DRP1-S616Ⓟ in situ.

### 3.4. Recombinant 3G11 Detected DRP1-S616 Phosphorylation Correlates with BRAF^V600E^ Disease

To explore the potential of recombinant 3G11 for immunohistochemistry (IHC) based applications, we evaluated DRP1-S616Ⓟ detection in formalin-fixed, paraffin-embedded (FFPE) human tissue samples. Prior work in melanoma showed a link between DRP1 status and BRAF^V600E^-driven disease [9,18]. Therefore, we aimed to determine whether 3G11 supports this association in patient samples.

We analyzed de-identified FFPE tissue sections obtained from the Mount Sinai Tisch Cancer Center Biorepository Shared Resource, including normal skin, benign nevi, atypical nevi, and primary melanomas. Immunohistochemistry was performed using the recombinant 3G11 and the BRAF^V600E^ mutant-specific antibodies, and staining intensity was compared across lesion types. Normal skin samples served as a negative control for both DRP1-S616Ⓟ (0 positive samples/23 total samples) and BRAF^V600E^ (0 positive samples/23 total samples) stainings and representative images are provided (Figure S2).

In benign nevi, 18/23 DRP1-S616Ⓟ positive samples (78.26%) were BRAF^V600E^ mutant; BRAF^Wt^ and BRAF^V600E^ lesions were 41.7% (5/12) and 46.2% (21/39) DRP1-S616Ⓟ positive, respectively (Table 2). Among atypical nevi with the BRAF^V600E^ mutation, 17/21 samples (80.95%) were DRP1-S616Ⓟ positive; BRAF^Wt^ and BRAF^V600E^ lesions were 50% (6/12) and 81% (17/21) DRP1-S616Ⓟ positive, respectively (Table 3). Lastly, in the melanoma cohort, which included 24 BRAF^Wt^ and 57 BRAF^V600E^ tumors, we detected DRP1-S616Ⓟ in 45 samples; BRAF^Wt^ and BRAF^V600E^ lesions were 20.8% (19/24) and 70.2% (40/57) DRP1-S616Ⓟ positive, respectively (Table 4). These data suggest that DRP1-S616Ⓟ may be associated with clinically dysplastic lesions and primary melanoma, implicating chronic mitochondrial division as a potential mechanistic contributor to disease. Validations in larger, well-annotated cohorts are required to confirm this association.

## 4. Discussion

In this study, we developed and characterized 3G11, a recombinant monoclonal DRP1-S616Ⓟ antibody. We describe that 3G11 is a sensitive and reliable tool for detecting DRP1-S616Ⓟ across multiple applications. 3G11 exhibits high specificity for recombinant DRP1-S616Ⓟ protein (Figure 2), successfully detects endogenous DRP1-S616Ⓟ in cell lysates and isolated organelles via Western blot (Figure 2), and effectively tracks dynamic changes in DRP1-S616Ⓟ in response to oncogenic MAPK inhibition using immunofluorescence (Figure 3). Most notably, in patient tissue sections, 3G11 produced consistent staining patterns and revealed an association between DRP1-S616Ⓟ and BRAF^V600E^ in malignant primary melanocytic lesions (Table 2, Table 3 and Table 4). In studies we could not readily detect murine or rat forms of DRP1 with 3G11, nor did we obtain successful immunoprecipitation of human DRP1-S616Ⓟ.

Recombinant 3G11 readily detects DRP1-S616Ⓟ in archival and recent FFPE tissues, immunoblots, and immunofluorescence assays. This versatility expands the tools available for studying mitochondrial dynamics in diverse cancer models and human samples. This supports explorations aimed at understanding how DRP1-S616Ⓟ influences metabolic rewiring, therapy responses, and disease progression as chronic mitochondrial division is implicated in the mechanistic control of these phenotypes [9,21,22,23,24,25,26,27]. DRP1-S616Ⓟ status is also linked to tumor types beyond melanoma, so broader applications of 3G11 have the potential to define shared mitochondrial vulnerabilities in multiple tumor types [10,11,12,13,14,15]. The performance of 3G11 in these models remains to be established and warrants future study. Additionally, further investigations should also examine DRP1-S616Ⓟ in receptor tyrosine kinase (e.g., EGFR^L858R^) and RAS (e.g., N-RAS^Q61R^) mutant tumors to define a more complete biological and translational scope.

The scoring of the IHC sections was performed in a binary manner, consistent with the approach described in Weider et al. [18], and as the cohort size increased, this classification aligned with BRAF^V600E^ staining patterns. However, this approach does not capture potential staining heterogeneity, and future studies should incorporate cohort-level, semi-quantitative analyses to better resolve variation in signal intensity. Furthermore, the associations observed in Table 2, Table 3 and Table 4 vary across lesion types. In benign and atypical nevi, DRP1-S616Ⓟ positivity was not significantly associated with BRAF^V600E^ status, whereas a significant association was observed in primary melanoma (*p* < 0.0001).

We posit these findings are consistent with the established role of oncogene-induced senescence in early melanocytic lesions, where BRAF^V600E^ is present, but MAPK signaling remains constrained. As a result, activation of DRP1-S616 phosphorylation may be driven by multiple alternative kinases, diluting any direct association with BRAF status. In contrast, progression to melanoma is associated with escape from senescence and reactivation of MAPK signaling, which is linked to increased DRP1-S616 phosphorylation and its detection by 3G11. Indeed, melanoma cells may have greater reliance on mitochondrial dynamics to support proliferation, survival, and stress adaptation; as such, DRP1-mediated fission may become more directly linked to oncogenic signaling outputs. Thus, in primary melanoma, BRAF^V600E^-driven signaling more consistently promotes DRP1-S616 phosphorylation to meet metabolic and apoptotic thresholds, whereas in nevi, mitochondrial dynamics are less tightly coupled to oncogenic BRAF status and remain more heterogeneous [28,29].

These IHC patterns also suggest that DRP1-S616Ⓟ is more reflective of established malignant signaling rather than an indicator of early lesion initiation. Accordingly, the current data support the use of 3G11 as a tool to assess MAPK pathway activity in melanoma, while its prognostic value in benign or premalignant lesions remains unclear. Determining whether DRP1-S616Ⓟ provides information independent of BRAF^V600E^ status will require further evaluation in larger, clinically annotated cohorts. We also hypothesize that understanding the relationship between BRAF^V600E^ and DRP1-S616Ⓟ may reveal insights into mitochondria-focused metabolic vulnerabilities and stress signaling that could impact combination therapies and immune checkpoint inhibition.

Similar observations have been described in RAS-driven systems, which share features such as increased glycolytic activity and altered mitochondrial dynamics [9,10]. These observations support the use of DRP1-S616Ⓟ as a molecular readout of mitochondrial division in oncogenic contexts, although further validation across tumor types is needed, including brain, colon, lung, and pancreatic tumors, where DRP1 is implicated in disease [10,11,12,13,14,15]. Building upon this, the 3G11 antibody recognizing active DRP1 enables the integration of DRP1-S616Ⓟ into established biomarker paradigms, positioning mitochondrial dynamics alongside established melanoma markers to potentially support both discovery efforts and clinically actionable patient stratification, prognosis, and therapeutic response prediction [30,31].

Compared with most commercially available DRP1-S616Ⓟ antibodies commonly used in the literature, the new recombinant monoclonal antibody derived from a human DRP1 peptide offers several important advantages for the study of mitochondrial division. Most antibodies targeting DRP1-S616Ⓟ are polyclonal preparations generated against undefined phosphopeptides using standard hybridomas, resulting in heterogeneous antibody populations with variable affinity and epitope recognition. This heterogeneity can lead to lot-to-lot variability, reduced reliability, and increased susceptibility to cross-reactivity with closely related phospho-epitopes. In contrast, recombinant production of a defined VH/L human-sequence monoclonal DRP1-S616Ⓟ antibody ensures molecular uniformity, batch-to-batch consistency, stable long-term availability throughout research program longevity, and is more economical. The single human-specific epitope further improves performance in complex human samples, including FFPE tissues, where polyclonal reagents frequently exhibit elevated background or inconsistent staining and are not recommended. In summary, recombinant 3G11 demonstrates robust and reproducible detection of DRP1-S616Ⓟ across immunoblotting, immunofluorescence, and histologic platforms, thereby enhancing experimental rigor throughout a complex study and supporting its use for integrated mechanistic and translational investigations of mitochondrial division in cancer and related disease contexts.

## 5. Conclusions

This work introduces 3G11, a recombinant monoclonal antibody that detects DRP1-S616Ⓟ with high sensitivity and specificity across multiple experimental applications. 3G11 performed robustly in immunoblotting, immunofluorescence, and FFPE tissue sections. Using this reagent, we identified a consistent association between DRP1-S616Ⓟ and BRAF^V600E^ across benign, atypical, and malignant melanocytic lesions. These findings support prior reports linking oncogenic MAPK signaling to sustained DRP1-S616Ⓟ and underscore the relevance of this phosphorylation event as a molecular feature of oncogenic MAPK-driven tumors. Collectively, 3G11 represents a specific, versatile, renewable, and cost-effective tool for studying mitochondrial division, with strong potential for translational and clinical applications.

## Figures and Tables

**Table 1:** qPCR primer sequences for indicated genes.

Gene	Forward Primer (5′–3′)	Reverse Primer (5′–3′)
*18S*	TCACCCACACTGTGCCCATCTACG	CAGCGGAACCGCTCATTGCCAATGG
*DNM1L*	CATTGCTGACAGGATGCAGAAGG	TGCTGGAAGGTGGACAGTGAGG

**Table 2:** Recombinant 3G11 staining results with benign nevi sections. Immunohistochemistry was performed to detect the status of BRAF^V600E^ and DRP1-S616Ⓟ of benign nevi (51 samples). Fisher’s Exact and Chi-Squared tests determined statistical significance.

BRAF Status	DRP1-S616Ⓟ Status	Benign Nevi #/51 (%)	Fisher’s Exact	Chi-Squared (X^2^)
BRAF^Wt^	DRP1-S616Ⓟ (−)	7 (14%)	*p* = 0.9999	*p* = 0.7847
BRAF^Wt^	DRP1-S616Ⓟ (+)	5 (10%)		
BRAF^V600E^	DRP1-S616Ⓟ (−)	21 (41%)		
BRAF^V600E^	DRP1-S616Ⓟ (+)	18 (35%)		

**Table 3:** Recombinant 3G11 staining results with atypical nevi sections. Immunohistochemistry was performed to detect the status of BRAF^V600E^ and DRP1-S616Ⓟ of atypical nevi (33 samples). Fisher’s Exact and Chi-Squared tests determined statistical significance.

BRAF Status	DRP1-S616Ⓟ Status	Atypical Nevi #/33 (%)	Fisher’s Exact	Chi-Squared (X^2^)
BRAF^Wt^	DRP1-S616Ⓟ (−)	6 (18%)	*p* = 0.1142	*p* = 0.0627
BRAF^Wt^	DRP1-S616Ⓟ (+)	6 (18%)		
BRAF^V600E^	DRP1-S616Ⓟ (−)	4 (13%)		
BRAF^V600E^	DRP1-S616Ⓟ (+)	17 (51%)		

**Table 4:** Recombinant 3G11 staining results with primary melanoma sections. Immunohistochemistry was performed to detect the status of BRAF^V600E^ and DRP1-S616Ⓟ of primary melanoma (81 samples). Fisher’s Exact and Chi-Squared tests determined statistical significance.

BRAF Status	DRP1-S616Ⓟ Status	Melanoma #/81 (%)	Fisher’s Exact	Chi-Squared (X^2^)
BRAF^Wt^	DRP1-S616Ⓟ (−)	19 (24%)	*p =* <0.0001	*p* = 0.0001
BRAF^Wt^	DRP1-S616Ⓟ (+)	5 (6%)		
BRAF^V600E^	DRP1-S616Ⓟ (−)	17 (21%)		
BRAF^V600E^	DRP1-S616Ⓟ (+)	40 (50%)		

## Data Availability

The raw data supporting the conclusions of this article will be made available by the authors on request.

## Supplementary Materials

The following supporting information can be downloaded at: https://www.mdpi.com/article/10.3390/antib15020038/s1. Figure S1: Dot blot screening of DRP1-S616Ⓟ anti-sera; Figure S2: Representative immunohistochemical stainings for DRP1-S616Ⓟ and BRAF^V600E^ in tissue cohorts; Excel File: 3G11 (and other candidate antibodies) heavy and kappa chain variable region amino acid sequences.

## Abbreviations

The following abbreviations are used in this manuscript:

DMSO	Dimethyl Sulfoxide
DRP1	Dynamin Related Protein 1
ERK1/2	Extracellular Regulated Kinase 1/2
IMM	Inner Mitochondrial Membrane
MAPK	Mitogen-Activated Protein Kinase
MFN1/2	Mitofusin-1/2
OIS	Oncogene-Induced Senescence
OMM	Outer Mitochondrial Membrane
OPA1	Optic Atrophy 1
ELISA	Enzyme-Linked Immunosorbent Assay
VH/L	Variable Heavy and Light Chains
