# Supplementary material for: A Recombinant Antibody Against Human DRP1 Serine 616 Phosphorylation Enables Detection of BRAFV600E-Associated Mitochondrial Division in Cancer
Source: Antibodies (Basel). 2026 Apr 20;15(2):38. doi: 10.3390/antib15020038 (PMC13113566; doi:10.3390/antib15020038)
Supplement: Supplementary file 1 [file antibodies-15-00038-s001.zip › antibodies-4115904-supplementary.pdf]

antibodies

MDPI

Supplementary Materials

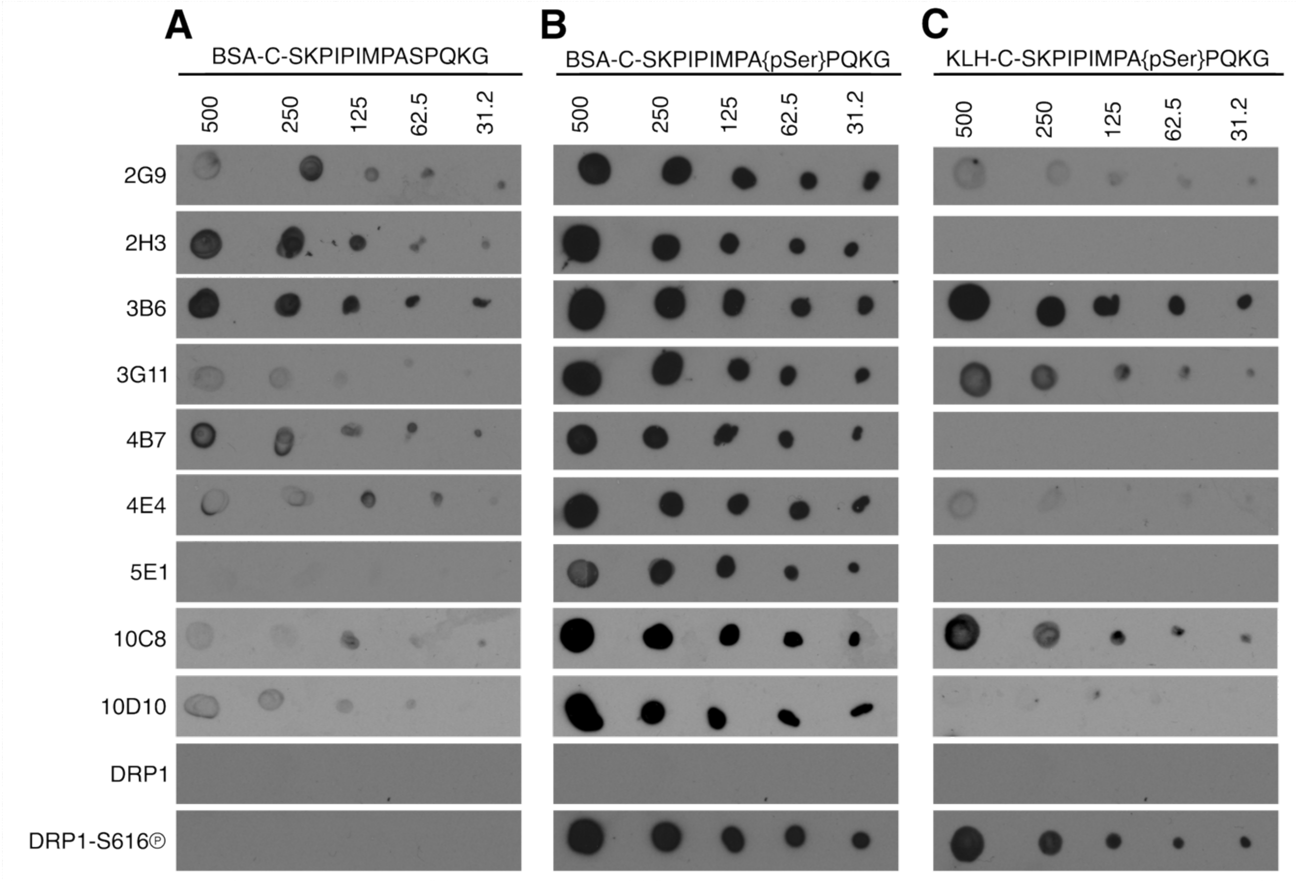

Figure S1. (Related to Figure 1). Dot blot screening of DRP1-S616Ⓟ anti-sera. (A–C) Dot blot analysis of anti-sera assessing specificity for the KLH-conjugated DRP1-S616Ⓟ peptide. Indicated peptides were spotted and dried onto nitrocellulose membranes at decreasing amounts (ng/dot) and detected by standard enhanced chemiluminescence. The bottom two rows of dot blots were probed using commercially available purified rabbit polyclonal DRP1 and DRP1-S616Ⓟ antibodies.

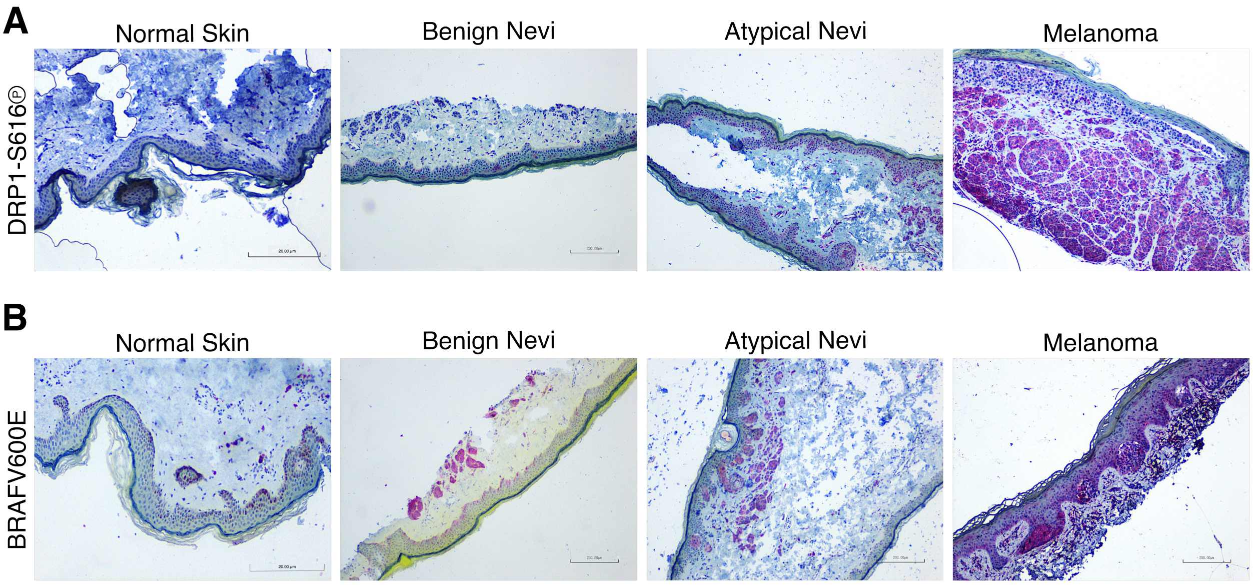

Figure S2 (Related to Tables 1–3). Immunohistochemical stainings for DRP1-S616℗ and BRAF^{V600E} in patient cohorts. (A) Representative images of recombinant 3G11 staining in normal skin, benign nevi, atypical nevi, and primary melanoma lesions. Normal skin serves as the control tissue and defines negative staining. Benign nevi show limited and diffuse staining. Atypical nevi show increased staining relative to benign nevi. Primary melanoma lesions show strong and diffuse staining. **(B)** Representative images of BRAF^{V600E} staining in normal skin, benign nevi, atypical nevi, and primary melanoma lesions. Normal skin serves as the control tissue and defines negative staining. Benign nevi showed broad BRAF^{V600E} positivity patterns including no staining, diffuse, and/or strong intensity; we presume due to the heterogeneous nature of benign nevi and a combination of lesions that were stable versus a set that potentially progressed to dysplastic and/or primary disease. Atypical nevi show increased staining compared to benign nevi. Primary melanoma lesions show robust cytosolic staining.
